# Transesterification of PHA to Oligomers Covalently Bonded with (Bio)Active Compounds Containing Either Carboxyl or Hydroxyl Functionalities

**DOI:** 10.1371/journal.pone.0120149

**Published:** 2015-03-17

**Authors:** Iwona Kwiecień, Iza Radecka, Marek Kowalczuk, Grażyna Adamus

**Affiliations:** 1 Centre of Polymer and Carbon Materials, Polish Academy of Sciences, Zabrze, Poland; 2 School of Biology, Chemistry and Forensic Science, Faculty of Science and Engineering, University of Wolverhampton, Wolverhampton, United Kingdom; Tsinghua University, CHINA

## Abstract

This manuscript presents the synthesis and structural characterisation of novel biodegradable polymeric controlled-release systems of pesticides with potentially higher resistance to weather conditions in comparison to conventional forms of pesticides. Two methods for the preparation of pesticide-oligomer conjugates using the transesterification reaction were developed. The first method of obtaining conjugates, which consist of bioactive compounds with the carboxyl group and polyhydroxyalkanoates (PHAs) oligomers, is "one-pot" transesterification. In the second method, conjugates of bioactive compounds with hydroxyl group and polyhydroxyalkanoates oligomers were obtained in two-step method, through cyclic poly(3-hydroxybutyrate) oligomers. The obtained pesticide-PHA conjugates were comprehensively characterised using GPC, 1H NMR and mass spectrometry techniques. The structural characterisation of the obtained products at the molecular level with the aid of mass spectrometry confirmed that both of the synthetic strategies employed led to the formation of conjugates in which selected pesticides were covalently bonded to PHA oligomers via a hydrolysable ester bond.

## Introduction

The targeted and controlled delivery of bioactive molecules is an area of considerable interest currently. Several approaches to this have been developed. Early controlled release methods involved bioactive compound-polymer physical formulations. Later developments included the preparation of covalently linked bioactive compound-polymer conjugates. Carriers for active compounds should be biodegradable and give non-toxic biodegradation products that are safe for the environment [1,2].

The replacement of conventional forms of pesticides with controlled-release formulations that show higher resistance to weather conditions is of interest. Polyhydroxyalkanoates have been examined as physical carriers and, for example, PHB-based polymeric matrices for Ronilan and Sumilex pesticides [3], poly(3-hydroxybutyrate-*co*-3-hydroxyvalerate) for sustained-release formulations of the herbicide Zellek Super in the form of films [4] or poly(3-hydroxybutyrate-*co*-3-hydroxyvalerate) microspheres loaded with atrazine herbicide [5] have been described. In these admixture systems, active compounds are mixed with polymers and are released over the course of several days. However, this period of time is brief compared with the length of the growing season. Thus, a major disadvantage of the admixture approach is that the release of the bioactive compound is largely dependent on the breakdown of the physical polymer structure. This results in poor control of the rate of release. However, PHAs are still regarded as an appropriate material for pesticide controlled-release systems. PHAs are produced by prokaryotes from renewable resources, such as carbohydrates, lipids or alcohols, and from a broad range of waste and surplus materials, such as glycerol from biodiesel production, protein hydrolysates, as well as meat and bone meal from slaughtering and rendering industries or molasses and starch from the sugar industry [6]. Methods of biosynthesis of polyhydroxyalkanoates, such as the diblock polymer of poly(3-hydroxypropionate)-block-poly(4-hydroxybutyrate) [7], poly(3-hydroxydodecanoate-*co*-3-hydroxydecanoate) [8], poly(3-hydroxynonanoate-*co*-3-hydroxyheptanoate) [9] and other medium chain length PHAs [10,11] have been developed dynamically. In addition, PHAs are currently produced on an industrial scale and are commercially available mostly for the production of compostable packaging [12]. Therefore, the price of PHA is expected to decrease with the increase of their production volume.

The development of PHA-based plant protection products via the linking approach requires efficient preparation methods of oligomers possessing biological efficacy. Recently, several of us reported the possibility of obtaining pesticide conjugates with a synthetic analogue of natural PHB (e.g. conjugates of (4-chloro-2-methylphenoxy)acetic acid (MCPA) or (2,4-dichlorophenoxy)acetic acid (2,4-D) with atactic oligo(3-hydroxybutyrate) (OHB)) via the anionic ring-opening oligomerisation of β-butyrolactone [13]. The effectiveness of selected conjugates obtained through ring-opening oligomerisation of racemic β-butyrolactone was confirmed using greenhouse tests [14,15]. The herbicidal effectiveness of the amorphous 3-HBA oligomers was comparable to that of DMA salts of 2,4-D and dicamba in greenhouse and field bioassays.

In this study, the focus was shifted toward the synthesis of bioactive PHA conjugates from natural biopolyesters. To the best of our knowledge, pesticidal conjugates of natural PHAs have not been reported until now. We hypothesised that the bioactive compound could function as a substrate of the PHA transesterification reaction if it contains either carboxyl or hydroxyl functionalities. Furthermore, the introduction of biologically active compounds to the chains of commercially available PHA by a relatively simple procedure will allow for development of economically favourable, controlled release systems. Herein, we present two methods for the preparation of delivery systems of selected bioactive compounds (in particular pesticides) based on the transesterification reactions of PHA biopolyesters. The first method is a one-pot synthesis in which the respective pesticide-oligomer conjugates were obtained through a transesterification reaction of PHA biopolyesters by selected pesticide with the carboxyl group in the presence of 4-toluenesulfonic acid monohydrate (TSA · H_2_O).

The second method is a two-step procedure, in the first step, macrolides were obtained from PHB biopolyester according to the method described in the literature [16], in second step lipase-catalysed transesterification of the cyclic oligomers by pesticides leads to the formation of the conjugates. The transesterification reactions of poly(3-hydroxybutyrate) and poly(ε-caprolactone) [17] or poly(3-hydroxybutyrate) and poly(L-lactide)[18] catalysed by TSA·H_2_O have been previously published. Information regarding the reactions of cyclic PHB oligomers with alcohols[19] in the presence of lipases has also been reported. We are now reporting examples of transesterification of PHA biopolyesters as a way for the preparation of delivery systems for selected pesticides. The methods developed herein are relatively simple and enable the synthesis of pesticide-oligomer conjugates with anticipated structures and molar masses. Furthermore, the detailed structural characterisation of the resulting conjugates was performed at the molecular level. The biodegradable controlled delivery systems of pesticides developed here should show prolonged activity and reduced adverse effects on the environment.

## Materials and Methods

### Materials

The poly(3-hydroxybutyrate) was prepared via waste oil fermentation at the University of Wolverhampton[20]; the number-average molar mass, as determined by GPC, was M_n_ = 300000 g/mol and the dispersity index was M_w_/M_n_ = 3.5. The poly(3-hydroxybutyrate-*co*-4-hydroxybutyrate) was purchased from Tianjin Green Bio-Science (Tianjin, China); the number-average molar mass, as determined by GPC, was M_n_ = 250000 g/mol and the dispersity index was M_w_/M_n_ = 2.5. The 4HB unit content was 8.8 mol% (based on the ^1^H NMR spectrum). Additionally, (4-chloro-2-methylphenoxy)acetic acid, dichloromethane, hexane and toluene were supplied by POCH S.A (Gliwice, Poland). The 4-(2-hydroxyethyl)phenol (tyrosol), lipase acrylic resin from *Candida antarctica* (≥10,000 U/g, recombinantly expressed in *Aspergillus niger* (*CA*)) and 4-toluenesulfonic acid monohydrate were purchased from Sigma-Aldrich Chemie GmbH (Steinheim, Germany).

### Physicochemical characterisation

#### Gel permeation chromatography (GPC) analysis

The number-average molar mass (M_n_) and the dispersity index (M_w_/M_n_) of the samples were determined by GPC. The GPC analyses were run using Viscotek VE 1122 pump, Shodex SE-61 RI detector, PLgel 3 μm MIXED-E (Polymer Laboratories) high-efficiency column (300 mm × 7.5 mm). The analysis were performed at 35°C using CHCl_3_ as eluent at the flow rate 1 mL/min. The instrument was calibrated with polystyrene narrow standards.

#### Nuclear magnetic resonance (NMR) analysis

^1^H NMR spectra were run in CDCl_3_ with tetramethylsilane as the internal standard using a Bruker Ultrashield AVANCE II 600 MHz spectrometer (Bruker BioSpin GmbH, Rheinstetten, Germany).

#### Electrospray mass spectrometry (ESI-MS^n^) analyses

For electrospray mass spectrometry analyses, a Finnigan LCQ ion-trap mass spectrometer (Finnigan, San Jose, CA, USA) was used. Solutions of samples (methanol/chloroform, 2:1 v/v) were introduced into the ESI source by continuous infusion with 3 μL/min flow rate using an instrument syringe pump. Settings and conditions: spray voltage: 4.5 kV; capillary temperature: 200°C; the sheath gas: nitrogen; auxiliary gas: helium. ESI-MS/MS experiments: precursor ions, isolated by ion trap, were collisionally activated. The RF amplitude was set to a value providing the decrease of the peak height of the fragmented ion by at least 50%. The analyses were performed using positive-ion mode.

### Synthesis

#### Preparation of pesticide-oligomer conjugates—one-step method

Poly(3-hydroxybutyrate-*co*-4-hydroxybutyrate) (0.5 g) and an appropriate amount of MCPA and TSA · H_2_O were introduced into a round bottom flask equipped with a magnetic stirring bar, and the reaction vessel was placed into a Heat-On Block System (R.B.Radley Co. Ltd) located on a stirring hot plate. The reaction was performed under an argon atmosphere at 158–163°C. The molten reagents were stirred for two minutes, then the reaction mixture was cooled; 10 cm^3^ of dichloromethane was added, and the organic layer was washed five times with distilled water to remove any residual 4-toluenesulfonic acid. The products were precipitated with cold hexane and dried under a vacuum at room temperature.

#### Preparation of pesticide-oligomer conjugates—two-step method

Cyclic oligomers were prepared according to the method developed by Seebach et al [16]. 30 mg of cyclic oligomers, 150 mg of lipase from *Candida antarctica* immobilised on acrylic resin, 25 mg of tyrosol and 12 cm^3^ of toluene were added to a round bottom flask equipped with a magnetic stirring bar. The flask was placed into a heated bath, and the reaction mixture was stirred under an argon atmosphere at 45°C for 120 h. The enzymes were removed by filtration, and the solvent was evaporated under reduced pressure.

### Hydrolytic degradation

Studies of hydrolytic degradation of MCPA–oligo(3HB-*co*-4HB) conjugates were performed under laboratory conditions. Glass vials containing samples of MCPA–oligo(3HB-*co*-4HB) conjugates (20 mg) and deionised water (10 cm^3^) were placed in a thermostatically controlled incubator set at 25°C. Vials with samples were withdrawn in triplicate from the incubator after 1, 3, 6, 9, 12, 16 and 20 weeks; samples were analysed using ESI-MS technique.

## Results and Discussions

In our previous studies we synthesised bioactive conjugates via anionic ring opening polymerisation (ROP) of 4-metyl-2-oxetanone using the activated salts of bioactive compounds as an initiator. However, this method was limited only to bioactive compounds possessing carboxyl functionality. Moreover, due to the special requirements associated with the purity that must be fulfilled by solvent and monomer in the anionic ROP method, the scaling-up the synthesis of pesticide-polymer conjugates may be troublesome. Therefore, we have currently focused our research on the development of a pesticide delivery system based on the transesterification reaction of natural PHA. It is worth noting, that oligomers formed from a polymeric carrier during the action of the delivery system reported here, will not be harmful to the environment because PHA biopolyesters are biodegradable and the products of their degradation are bioassimilated by various bacteria strains.

### Synthesis of MCPA–oligo(3HB-co-4HB) conjugate using the one-pot method

**Fig 1:**
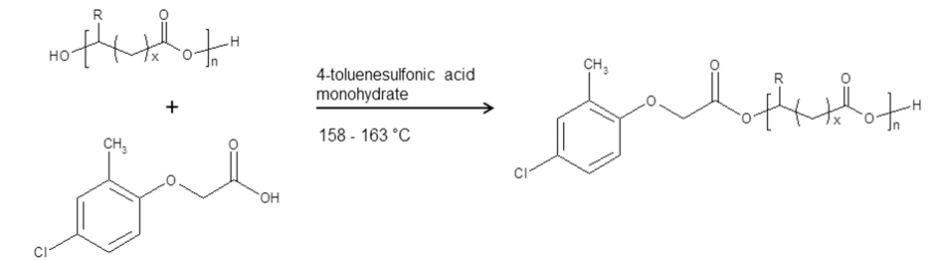
Transesterification reaction of P(3HB-co-4HB) by MCPA, mediated by TSA · H2O. R = CH_3_, x = 1–3HB units; R = H and x = 2–4HB units.

The synthesis of MCPA–oligo(3HB-*co*-4HB) conjugate was performed through a transesterification reaction of poly(3-hydroxybutyrate-*co*-4-hydroxybutyrate), (P(3HB-*co*-4HB)) biopolyester with (4-chloro-2-methylphenoxy)acetic acid (MCPA) in the presence of 4-toluenesulfonic acid monohydrate (Fig. 1).

The synthesis was carried out in one pot, without solvent, under an argon atmosphere. Our previously published studies on the synthesis and preliminary applications of pesticide–atactic oligo(3HB) conjugates showed that the 3-hydroxybutyrate oligomers with an average M_n_ of approximately 1000 g/mol gave the most promising results as polymer carriers in pesticide controlled-release systems [15]. The TSA · H_2_O introduced the water necessary for the partial hydrolysis of the P(3HB-*co*-4HB) biopolyester and the formation of biopolyester oligomers with hydroxyl and carboxyl end groups, which then undergo transesterification with the bioactive compounds [17]. Based on our previous studies [18], available literature data and on preliminary experiments performed, the reaction conditions i.e. time, temperature as well as the amounts of biopolyester, catalyst and biologically active substances were matched in such a way that the molar masses of the resulting pesticide-oligomer conjugates were approximately 1000 g/mol. The temperature and time of transesterification reaction were determined experimentally at 160°C for 2 minutes. Conducting of the transesterification process under such conditions allows complete melting of P(3HB-*co*-4HB) with minimal share of side reaction, such as the thermal degradation process of P(3HB-*co*-4HB) which leads to formation of (3HB-*co*-4HB) oligomers with crotonate end-groups. At the same time this allows us to obtain a high level of attachment of MCPA to (3HB-*co*-4HB) oligomer. It was also experimentally observed that there was a relationship between average molar masses (M_n_) of the resulting conjugates and the amount of TSA · H_2_O used in the process. The increase of the quantity of water, which was introduced with TSA, led to the decrease of molar mass of resulting oligomers. Finally, in order to develop optimal conditions of transesterification, the ratio of biopolyester/MPCA/TSA · H_2_O was experimentally determined. For this purpose, while maintaining in all experiments a constant amount of the biopolyester P(3HB-*co*-4HB), the amounts of MPCA and TSA · H2O were matched in weight percent compared to the biopolyester. The results of the syntheses are summarised in Table 1. Each experiment presented in the Table 1 was repeated three times.

**Table 1:** Results of the transesterification reaction of P(3-HB-*co*-4HB) by MCPA in the presence of 4-toluenesulfonic acid monohydrate (TSA · H_2_O).

sample	MCPA(wt %)*	TSA · H_2_O(% wt.)	M_n_[g/mol]^a^	M_w_/M_n_ ^a^	The amount of MCPA attached to the 3HB-*co*-4HB oligomers in [mol%]^b^
1	40	10	1400	3,1	39±4
2	40	15	1700	2,4	45±3
3	40	20	900	1,8	73±2
4	40	25	900	1,8	74±2
5	40	40	700	1,5	85±2
6	60	10	1100	1,3	18±3
7	90	10	1200	1,5	15±3
8	60	20	900	1,8	69±2

^a^Determined by GPC,

^b^molar percentage of pesticide attached to the oligomer chains, calculated from the ^1^H NMR spectra.

* The amount of MCPA compared to that of P(3HB-*co*-4HB) used for the transesterification reactions, expressed in weight percent.

P(3HB-*co*-4HB), M_n_ = 250000 g/mol and the dispersity index M_w_/M_n_ = 2.5, 8.8 mol% of the 4HB unit content.

The resulting products were characterised using gel permeation chromatography (GPC), nuclear magnetic resonance (NMR) and electrospray ionisation mass spectrometry (ESI-MS).

**Fig 2:**
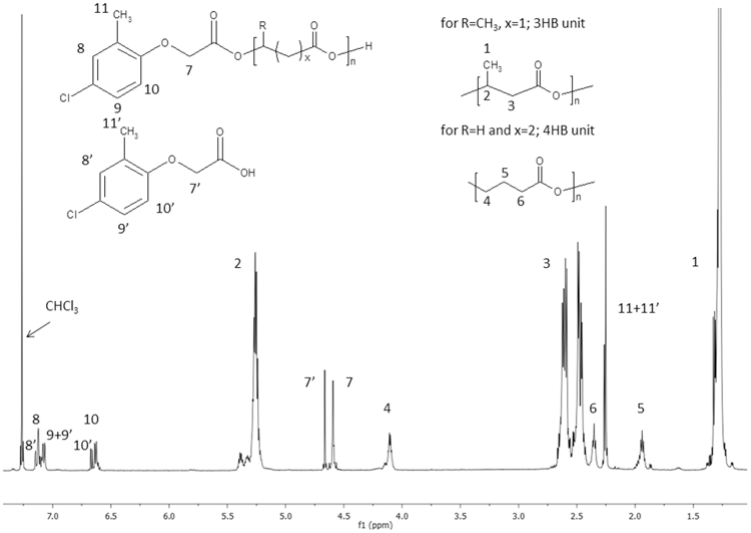
The ^1^H NMR spectra of sample 3 from Table 1.

The structural characterisation of the resulting products was performed with the aid of ^1^H NMR analyses. The ^1^H NMR spectrum of the products obtained from the reaction of MCPA and P(3HB-*co*-4HB) mediated by TSA · H_2_O (sample 3, Table 1) displayed signals corresponding to the protons of the 3-hydroxybutyrate repeating units (signals1–3) and signals corresponding to the protons of the 4-hydroxybutyrate repeating units (signals 4–6), as shown in Fig. 2. It is important to note that the content of the 4HB units in the transesterification product correlates to that of unmodified P(3HB-*co*-4HB) and was equal to approximately 8.8 mol%. Moreover, the signals attributed to the protons of the pesticide bonded to the oligomers (7–11), as well as signals of the protons of the unbonded pesticide (7’-11’), were also observed. Additionally, low-intensity signals that correspond to protons of the C**H**_3_- from crotonate end group at δ = 1.87 were detected.

The amount of pesticide attached to the PHA oligomers was calculated by analysing the intensities of the ^1^H NMR signals corresponding to protons of the—OC**H**_2_—groups of bonded MCPA (δ = 4.59, signal 7) and unbonded MCPA (δ = 4.67, signal 7’).

Previously published results concerning the transesterification reactions of poly(3-hydroxybutyrate) and poly(ε-caprolactone) catalysed by TSA · H_2_O revealed the presence of copolyester macromolecules that incorporated TSA as end groups [17]. Thus, the resulting transesterification products of P(3HB-*co*-4HB) with MCPA in the presence of TSA · H_2_O were examined for the presence of the polyester macromolecules terminated by the tosyl end group. For this purpose, the ^1^H NMR spectra of selected samples from Table 1 were compared with the corresponding ^1^H NMR spectra of the samples into which the TSA · H_2_O was added.

**Fig 3:**
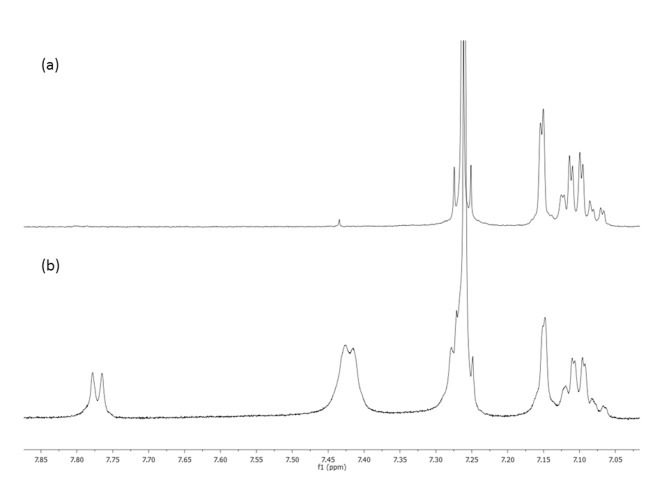
^1^H NMR spectra of sample 6 from Table 1 (a) and sample 6 with added 4-toluenesulfonic acid monohydrate (b).

Fig. 3 shows the ^1^H NMR spectrum of sample 6 (Table 1). Signals for the aromatic protons of the tosyl groups were not observed (Fig. 3a). However, when TSA · H_2_O was added, the aromatic proton signals of the tosyl group were observed at δ = 7.42 and δ = 7.77 (Fig. 3b). Thus, ^1^HNMR analysis confirmed that the resulting transesterification products did not contain oligomers with tosyl end groups.

^1^H NMR analysis confirms that it is possible to introduce pesticide molecules onto the oligo(3HB-*co*-4HB) chains using the one-pot synthesis method. It is worth mentioning that some unbonded pesticides remain in the product mixtures after purification due to their low water solubility; MCPA water solubility is 640 mg/L [21]. However, the presence of unbonded pesticide does not influence the effectiveness of the developed systems.

Inspection of Table 1 shows that the amount of pesticide attached to the P(3HB-*co*-4HB) oligomers depends on the amount of TSA · H_2_O used. Samples 1–8 from Table 1 were obtained through the reaction of MCPA with P(3HB-*co*-4HB) (Fig. 1). Samples 1–5 (Table 1) were synthesised via the transesterification reactions of P(3HB-*co*-4HB) by MCPA (40 wt. %) in the presence of TSA·H_2_O from 10 to 40 wt. %. The mol % of MCPA bonded to the P(3HB-*co*-4HB) oligomers increased as the amount of TSA · H_2_O increased. Samples 6 and 7 (Table 1) were obtained in the presence of 60 or 90 wt. % MCPA and were mediated by 10 wt. % TSA · H_2_O. In these cases, less than 20 mol % pesticide was attached to the oligomer. Based on these results, it was determined that the use of large amounts of pesticides in reactions mediated by small amounts of TSA · H_2_O (10 wt. %) was not justified.

The highest amount of MCPA attached to oligomers obtained from P(3HB-*co*-4HB) was achieved for sample 5 (in a reaction mediated by 40 wt. % TSA · H_2_O). However, it is preferable to use not more than 20 wt. % of TSA · H_2_O because increasing the quantity of TSA · H_2_O by 5% negligibly changed the amount of attached pesticide (see samples 3 and 4, Table 1), and even doubling the quantity of TSA · H_2_O from 20 to 40 wt. % resulted in only a slight increase in the amount of attached MCPA (see samples 3 and 5, Table 1).

Summarising the results in Table 1, 20 wt. % TSA · H_2_O is optimal for the synthesis of the MCPA–oligo(3HB-*co*-4HB) conjugate via transesterification.

**Fig 4:**
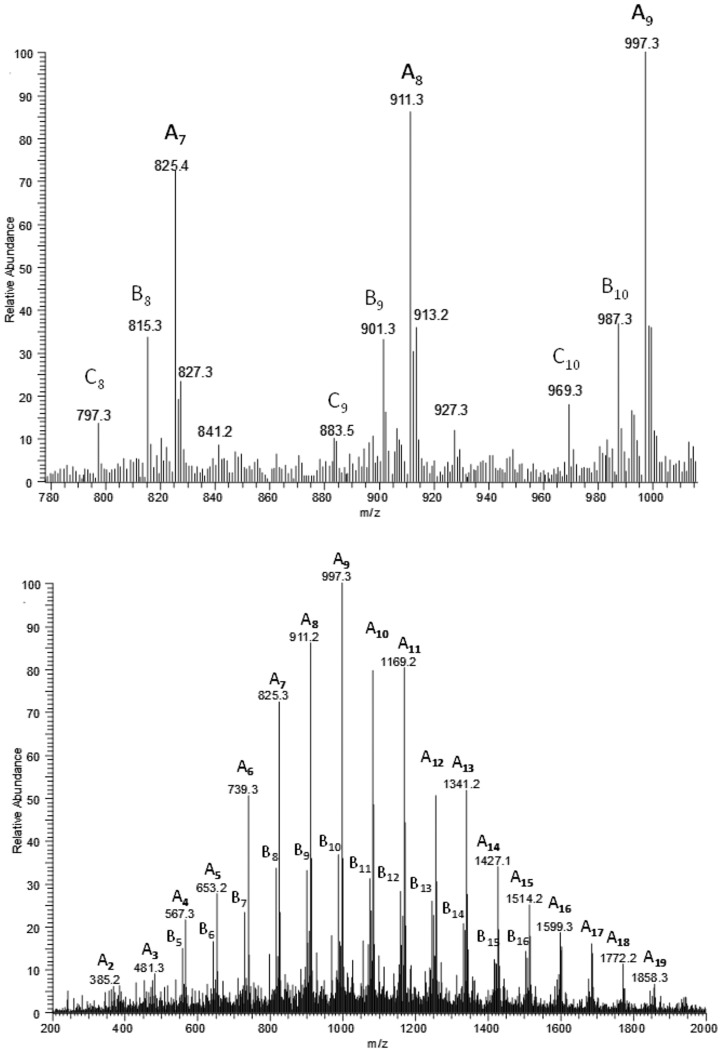
ESI-MS spectrum in positive-ion mode of the pesticide-oligomer conjugates obtained by transesterification reaction between P(3HB-*co*-4HB) and MCPA and spectral expansion in the range *m/z* 780–1020.

**Fig 5:**
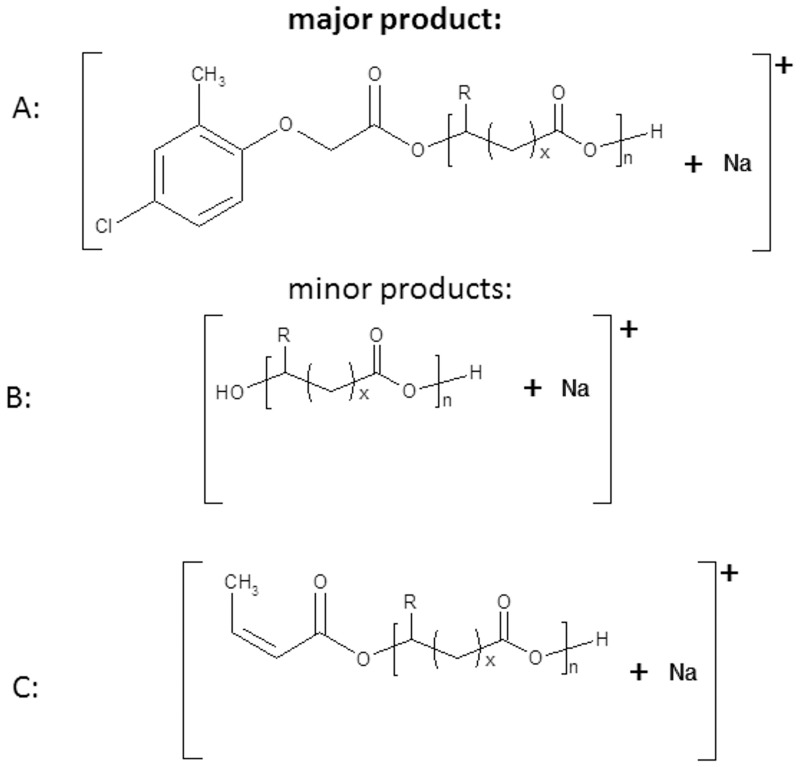
Structures of the ions present in the ESI-MS spectrum in Fig. 3 (for 3HB units R = CH_3_, x = 1, for 4HB units R = H and x = 2).

Advanced molecular characterisation of oligomers was supported by ESI-MS and ESI-MS^n^ analysis. Recently, ESI-MS has been successfully applied by our group in the structural characterisation of copolyesters obtained via the anionic ring-opening copolymerisation of β-butyrolactone with β-substituted β-lactones, such as β-ethoxymethyl-β-propiolactone [22] or β-butoxymethylpropiolactone, β-phenoxymethylpropiolactone and β-benzoxymethylpropiolactone [23], as well as atactic conjugates obtained by ROP: ibuprofen-oligo(3-hydroxybutyrate) conjugates [24], lipoic acid-oligo-(3-hydroxybutyrate) conjugates [25], MPCA-oligo-(3-hydroxybutyrate) conjugates [13] and food preservative–oligo(3-hydroxybutyrate) conjugates [26]. It was also successfully used for the characterisation of oligodiols obtained from atactic poly(3-hydroxybutyrate) or bacterial poly(3-hydroxybutyrate-*co*-4-hydroxybutyrate) [27]. The positive ESI mass spectrum of the sample selected from Table 1 (sample 3) is presented in Fig. 4. The 3-hydroxybutyrate and 4-hydroxybutyrate units are indistinguishable by mass spectrometry because these two units have the same molar mass. The spectrum contains one main series of ions, labelled **A** (series of signals at *m/z* 200+(86×n)+23), which corresponds to the sodium adduct of the MCPA-oligo(3HB-*co*-4HB). Series **A** of ions contain a chlorine atom and is particularly easy to recognise because the presence of this atom results in a characteristic isotopic profile of molecular ions that cannot be mistaken for any other structure [13]. These characteristic isotopic profiles of the molecular ions in the mass spectra of compounds containing chlorine result from the presence of two stable isotopes of chlorine, ^35^Cl (75.77%) and ^37^Cl (24.23%). The other signals present in the ESI-MS spectrum (of significantly lower intensity) are assigned to the sodium adduct of oligo(3HB-*co*-4HB) with hydroxyl and carboxyl end groups (series of signals at *m/z* 104+(86×n)+23), labelled **B,** and the sodium adduct of oligo(3HB-*co*-4HB) with crotonate (from 3HB units) and carboxyl end groups (series at *m/z* (86×n)+23), labelled **C**. The structures of the ions are shown in Fig. 5. However, the formation of isobaric cyclic structures (proportional to the content of 4HB units in unmodified polymers) cannot be excluded.

The ESI-MS^n^ experiments have been used to verify the chemical structures of the end groups of the oligomers obtained via the TSA· H_2_O-mediated transesterification reaction of natural P(3HB-*co*-4HB) by MCPA (sample 3, Table 1). Previously conducted fragmentation studies of individual molecular ions of the P(3HB-*co*-4HB) copolyester as well as atactic oligo-3-hydroxybutyrate conjugates with selected bioactive compounds revealed that a random β-hydrogen rearrangement at the ester groups was the main mechanism inducing the fragmentation of the polyesters chains by ester bond cleavages [13,25–27].

**Fig 6:**
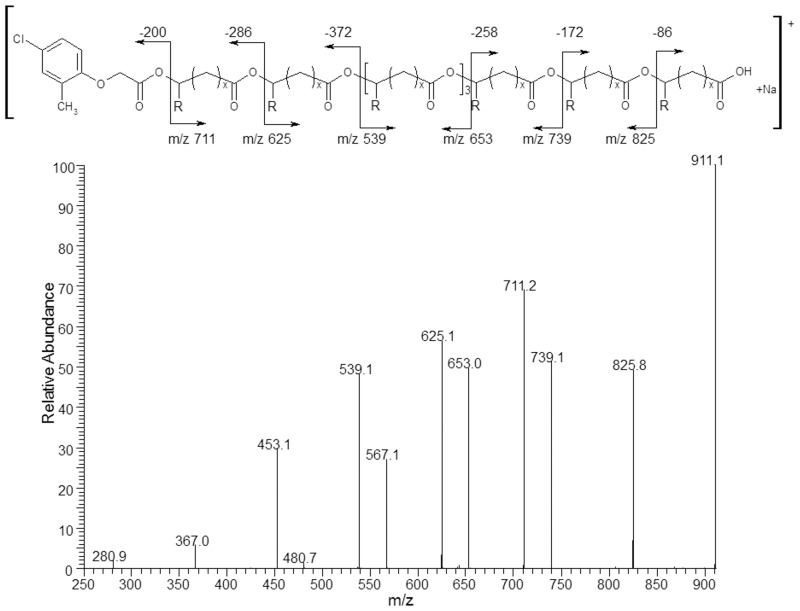
ESI-MS/MS product ion spectrum of the sodiated MCPA-oligo(3-hydroxybutyrate-*co*-4-hydroxybutyrate) conjugate at *m/z* 911.

Fig. 6 shows the results of an ESI-MS/MS experiment performed for the ion at *m/z* 911, which corresponds to the sodium adduct of MCPA-oligo(3HB-*co*-4HB) and is representative of series **A**, which was selected from the spectrum of sample 3 (Table 1, Fig. 3). According to the assigned structures, the product ions at *m/z* 825, 739, 653, 567 and 481 correspond to the oligo(3-hydroxybutyrate-*co*-4-hydroxybutyrate) terminated by the MCPA and carboxyl end groups. The complementary product ions at *m/z* 711, 625, 539, 453, 367 and 281 correspond to the oligo(3-hydroxybutyrate-*co*-4-hydroxybutyrate) with crotonate and carboxyl end groups. For example, the product ion at *m/z* 711 corresponds to the oligomer formed by the loss of the MCPA molecule (200 Da), and the product ion at *m/z* 825 corresponds to the oligomer formed by the loss of mainly crotonic acid (86 Da).

**Fig 7:**
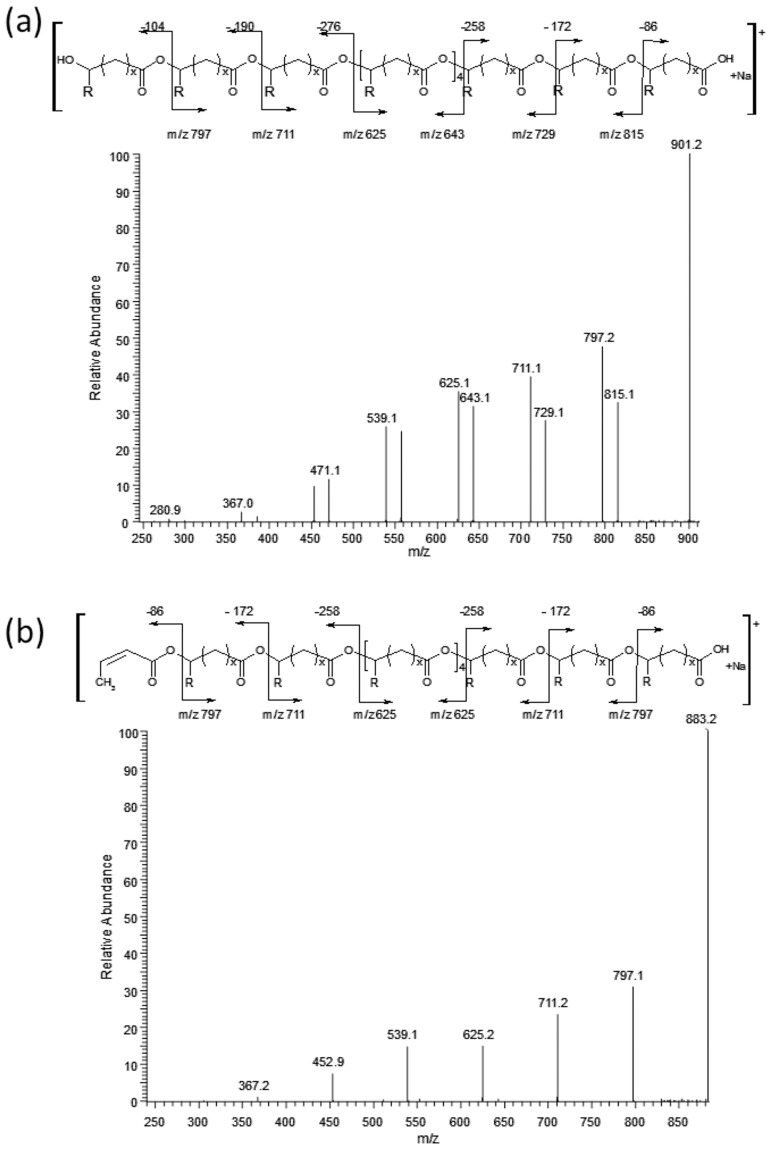
ESI-MS/MS product ion spectrum of the sodium adduct of oligo(3HB-*co*-4HB) with hydroxyl and carboxyl end groups at *m/z* 901 (a) and ESI-MS/MS product ion spectrum of the sodium adduct of oligo(3HB-*co*-4HB) with crotonate and carboxyl end groups at *m/z* 883 (b).

Fig. 7a shows the results of an ESI-MS/MS experiment performed for the ion at *m/z* 901, which corresponds to the sodium adduct of oligo(3HB-*co*-4HB) with hydroxyl and carboxyl end groups, selected from the ESI-MS spectrum of sample 3 (Table 1, Fig. 3). According to the assigned structures, the product ions at *m/z* 815, 729, 643, 557, 471 and 385 correspond to the oligo(3-hydroxybutyrate-*co*-4-hydroxybutyrate) with hydroxyl and carboxyl end groups. The complementary product ions at *m/z* 797, 711, 625, 539, 453, 367 and 281 correspond to the oligo(3-hydroxybutyrate-*co*-4-hydroxybutyrate) with crotonate and carboxyl end groups. For example, the product ion at *m/z* 797 corresponds to the oligomer formed by the loss of 3-hydroxybutyric acid (104 Da), and the product ion at *m/z* 815 corresponds to the oligomer formed by the loss of mainly crotonic acid (86 Da).

Fig. 7b shows the results of an ESI-MS/MS experiment performed for the ion at *m/z* 883 (selected from the ESI-MS spectrum in Fig. 3), which corresponds to the sodium adduct of oligo(3HB-*co*-4HB) with crotonate and carboxyl end groups. The fragmentation of this ion leads to the formation of only one set of oligo(3-hydroxybutyrate-*co*-4-hydroksybutyrate) fragment ions due to the identical molar masses of the 3HB and 4HB units.

Multistage electrospray ionisation mass spectrometry analyses were performed for all of the samples listed in Table 1.

The structural characterisation of the resulting products performed with the aid of the ESI-MS^n^ technique and supported by ^1^H NMR analysis indicated that the one-pot transesterification method of P(3HB-*co*-4HB) by active compounds mainly leads to (3HB-*co*-4HB) oligomers containing chemically bonded bioactive compounds. In addition to oligomers with pesticide end groups, small amounts of (3HB-*co*-4HB) oligomers terminated by hydroxyl and carboxyl, as well as with unsaturated and carboxyl end groups were identified. The (3HB-*co*-4HB) oligomers terminated by the hydroxyl and carboxyl end groups are formed due to the partial hydrolysis of the P(3HB-*co*-4HB) biopolyester in the presence of TSA · H_2_O in the reaction medium without subsequent transesterification by the bioactive compounds. The presence of (3HB-*co*-4HB) oligomers terminated by the crotonate end groups can be explained by the partial thermal degradation of P(3HB-*co*-4HB), which mainly occurs through a random chain scission mechanism due to the *β*-CH hydrogen transfer at the 3-hydroxybutyrate units [28]. Earlier studies indicated that the presence of these types of side products do not negatively affect crops during greenhouse and field bioassays [14,15].

### Synthesis of oligo(3HB)-tyrosol conjugates using the two-step method

**Fig 8:**
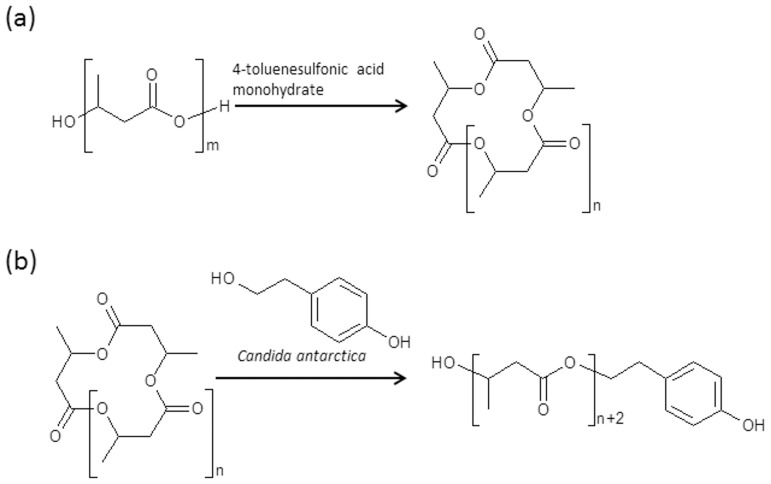
Ring-closing depolymerisation of PHB (a) and the reaction between cyclic oligo(3-hydroxybutyrate) and tyrosol (b).

The two-step approach was used to synthesise the oligo (3HB)-tyrosol conjugate. First, cyclic oligomers were obtained from PHB (Fig. 8a), and the cyclic oligomers were reacted with 4-(2-hydroxyethyl)phenol (tyrosol) in the presence of lipase (Fig. 8b). ESI-MS and ESI-MS^n^ were used to confirm the molecular structure of the resulting products.

**Fig 9:**
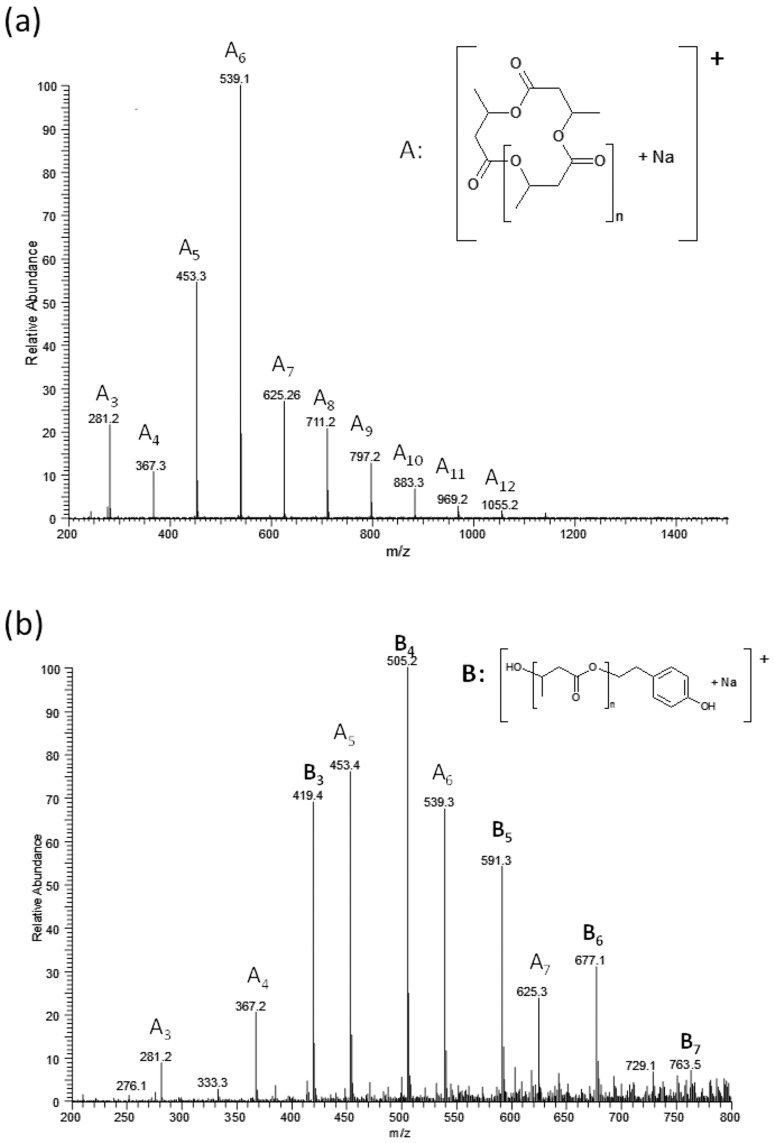
ESI-MS spectrum in positive-ion mode of cyclic oligomers (a) and ESI-MS spectrum in positive-ion mode of the pesticide-oligomer conjugates obtained from the reaction between cyclic oligomers and tyrosol (b).

Fig. 9a shows the ESI mass spectrum (positive-ion mode) of cyclic oligomers obtained in the ring-closing depolymerisation reaction of the poly(3-hydroxybutyrate) biopolyester (PHB) in solution in the presence of TSA · H_2_O. Fig. 9b shows the ESI mass spectrum of products obtained during the second step, i.e. after the reaction between cyclic oligo(3-hydroxybutyrate)s and tyrosol, which was carried out in hydrophobic solvents (e.g. toluene) in the presence of lipase from *Candida antarctica* immobilised on acrylic resin.

Along with signals corresponding to the conjugate oligomers, series **B** (Fig. 9b) signals corresponding to the sodium adduct of cyclic oligo(3-hydroxybutyrate) (series **A**) are present. These signals persist even after a significant increase in reaction time, which is associated with higher conversion.

**Fig 10:**
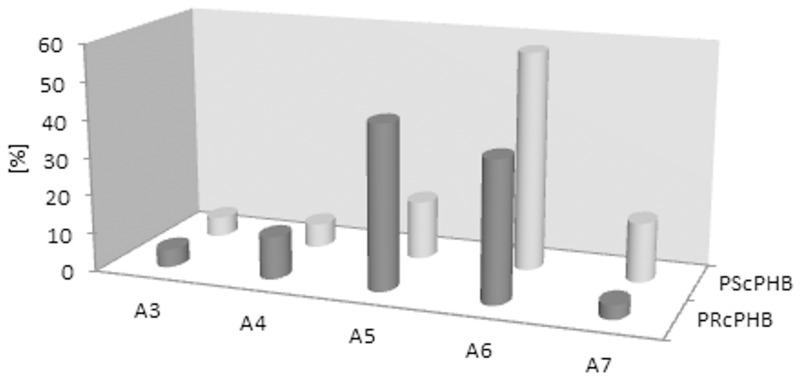
Population of the starting cyclic PHB oligomers (PScPHB) and population of the remaining cyclic PHB oligomers (PRcPHB).

It is known that in the case of applying the ESI-MS technique to the analysis of mixtures containing linear and cyclic oligomers that the intensities of the signals corresponding to the linear oligomers are lower than those of the cyclic ones. Cyclic oligomers are more easily ionisable; therefore, even a small amount of cyclic oligomer gives an intense signal. To quantitatively estimate the linear and cyclic oligomer content, separate calibrations for each type of oligomer would be necessary. However, the internal population of the cyclic oligomers before and after transesterification has been changed as presented in the bar chart (Fig. 10). These results have indicated that the higher cycles were the most reactive in the process studied, i.e. A6 and A7 (see Fig. 10).

The ESI-MS results confirmed that the conjugate molecules with the structures presented in Fig. 9b were formed using the two-step method described herein.

**Fig 11:**
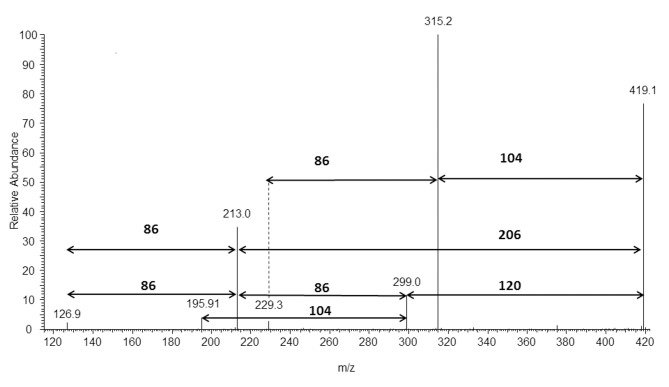
ESI-MS/MS product ion spectrum of sodiated tyrosol-oligo(3-hydroxybutyrate) conjugate at *m/z* 419.

**Fig 12:**
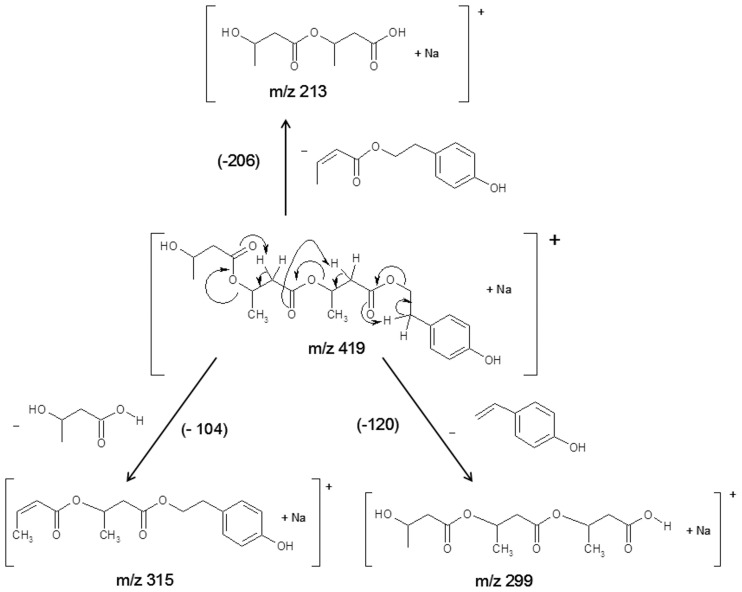
Fragmentation pathway and structures of the product ions formed during the ESI-MS/MS experiment of the sodiated tyrosol-oligo(3-hydroxybutyrate) conjugate at *m/z* 419.

Fig. 11 shows the results of an ESI-MS/MS experiment performed for the ion at *m/z* 419 (selected from the ESI-MS spectrum in Fig. 9b), which corresponds to the sodium adduct of oligo(3HB) with hydroxyl and tyrosol end groups. The β-hydrogen rearrangement at the ester groups was the main mechanism, similar as in our previous works [13,25–27], inducing the fragmentation of the oligomer chains by ester bond cleavages (see Fig. 12). The product ion at *m/z* 315 corresponds to the oligomer formed by the loss of 3-hydroxybutyric acid (104 Da), the product ion at *m/z* 299 corresponds to the oligomer formed by the loss of 4-ethenylphenol (120 Da) and the product ion at *m/z* 213 corresponds to the oligomer formed by the loss of 2-(4-hydroxyphenyl)ethyl crotonate (206 Da) (see Fig. 12).

To determine if both hydroxyl groups in tyrosol can react with cyclic oligo(3-hydroxybutyrate), a reaction between cyclic oligo(3-hydroxybutyrate) and phenol was performed under the same reaction conditions used for the reactions between cyclic PHA oligomers and tyrosol. In the positive ESI mass spectrum of the post-reaction mixture (data not shown), no conjugates of oligo(3-hydroxybutyrate) with phenol end groups were observed. Based on this result, it was assumed that cyclic PHA oligomers do not react with hydroxyl groups attached to a benzene ring in the presence of lipase from *Candida Antarctica*.

However, the two-step method of synthesis conjugates containing pesticides and PHA oligomers allowed us to obtain conjugates with more defined chain length than the first method.

Both synthetic strategies for preparation of delivery systems of pesticides reported here were based on the transesterification reaction of PHA with selected pesticides. In the first method, elaborated as "one-pot" method, reactions were carried out in the melt. The main drawback was the need for high temperature. The major advantages, however, were relatively short reaction time (2 minutes) and the eliminating of solvents during synthesis. The second method was the two-step method, with both steps carried out in organic solvents. The first step took 35 h and required temperature above 110°C to ensure reflux (by 20 h) and azeotropic water removal (by 15 h) [16]; the second step was carried out for 120 h in the presence of lipase. The second method was definitely more complicated and time-consuming than the first method, however, the population of bioactive oligomers obtained had more defined chain lengths.

### Release of pesticides from conjugates monitored by ESI-MS

Considering the perspective application of the obtained conjugates as release systems of pesticide with potential higher resistance to the weather conditions in comparison to the conventional forms of pesticides, the preliminary studies of the release of MPCA pesticide from MCPA—oligo(3HB-*co*-4HB) conjugate during incubation of this conjugate in water at the 25°C were performed. The hydrolytic degradation tests of MCPA—oligo(3HB-*co*-4HB) conjugate under laboratory condition was monitored with the aid of mass spectrometry.

**Fig 13:**
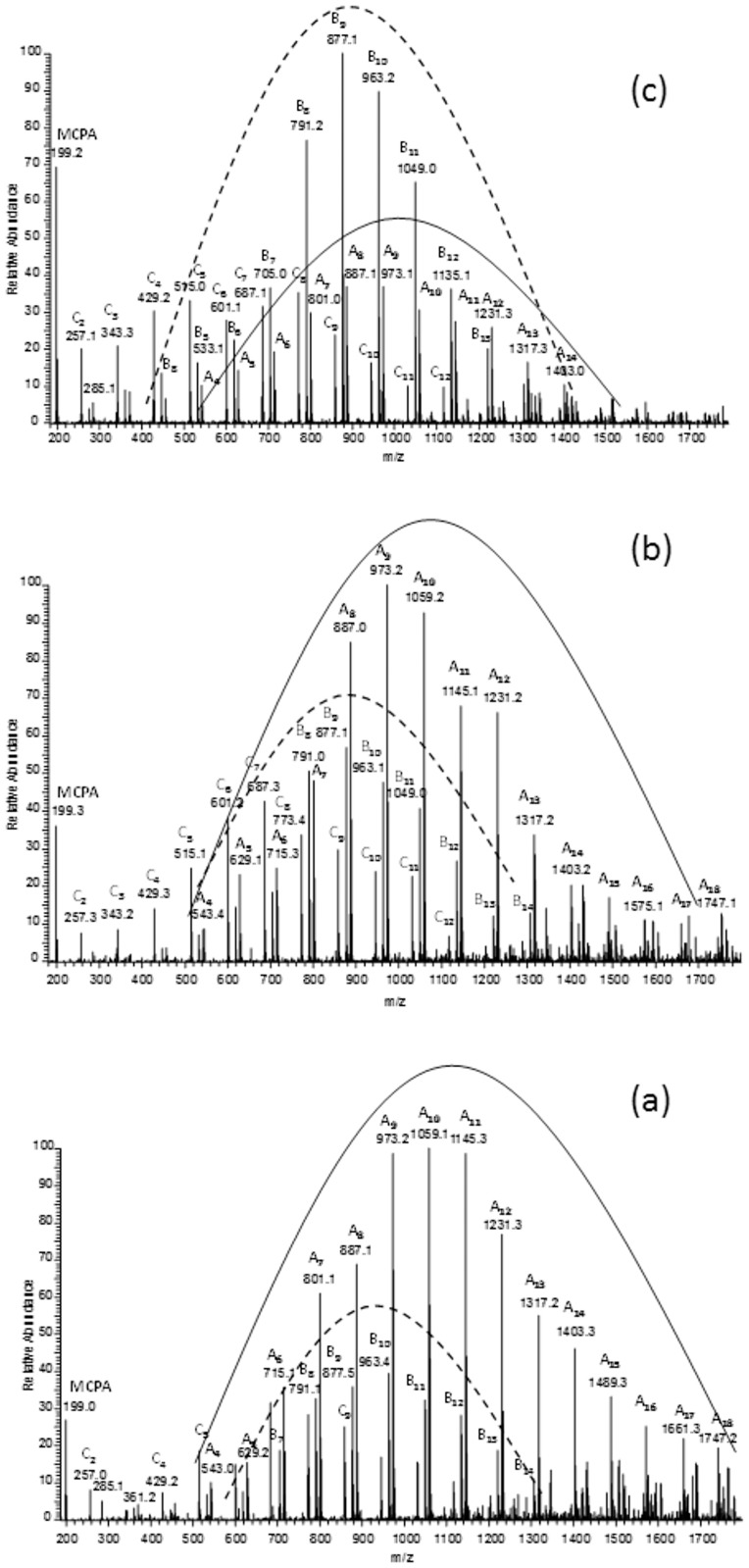
ESI-MS spectra in negative-ion mode of MCPA–oligo(3HB-*co*-4HB) conjugate before incubation (a), after 6 (b) and 20 (c) weeks of incubation of the conjugate in water at 25°C.

After different incubation times, the remaining conjugate samples were analysed by ESI-MS (negative-ion mode) to identify the released MCPA as well as (3HB-*co*-4HB) oligomeric degradation products. Fig. 13 shows negative ESI-MS spectra of conjugate before incubation time (Fig. 13a), after 6 weeks (Fig. 13b) and after 20 weeks (Fig. 13c) weeks of incubation of the conjugate in water at 25°C.

**Fig 14:**
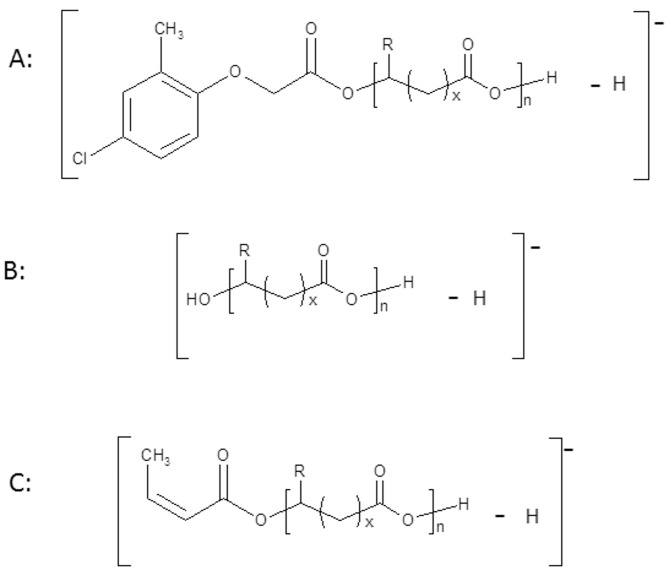
Structures of ions presented in the ESI-MS spectra in Fig. 9.

The mass spectrum of MCPA–oligo(3HB-*co*-4HB) conjugate acquired before incubation in water (Fig. 13a) contained three series of ions **A**, **B**, **C** as well as signal which corresponds to ion of free MCPA. The main series of signals at m/z 200+(86×n)-1 (labelled as **A** and marked by a solid line) was assigned to MCPA-oligo(3HB-co-4HB) conjugate chains. The other two series of signals (with significantly lower intensities) occurred at m/z 104+(86×n)-1 (dashed line) and at m/z 86+(86×n)-1, labelled as **B** and **C** correspond to individual oligo(3HB-*co*-4HB) chains terminated by hydroxyl and carboxyl end groups and with crotonate and carboxyl end groups, respectively. The structures of the signals present in the mass spectra in Fig. 13 are shown in Fig. 14. The spectra recorded after specific period of incubation in water showed that intensity of the signals corresponding to oligomers with lower molar masses increased as a function of hydrolysis time (Fig. 13b and c). Additionally, intensity of the signals which correspond to oligo(3HB-*co*-4HB) with hydroxyl and carboxyl end groups increased (series **B**, marked by dashed line). This supports the conclusion that the hydrolytic degradation process of P(3HB-*co*-4HB) progressed over the time, and after 20 weeks of degradation the **B** became main series in mass spectrum (Fig. 13c). Moreover, a high intensity signal at m/z 199 corresponding to free MPCA was observed, confirming the release of a MCPA moiety from the conjugate chain. The rate of hydrolytic degradation of MCPA–oligo(3HB-*co*-4HB) conjugates and release of pesticide from these conjugates under laboratory conditions did not reflect the degradation and release of pesticide under environmental condition. It is difficult to define the effects of all factors which exist in the environment. However, it is noteworthy that enzymes such as depolymerases, which cause PHA biodegradation, are secreted from a large variety of microorganisms widespread in natural environments [3,4] and these depolymerases may accelerate the release of pesticide from pesticide-PHA oligomer conjugates.

## Conclusions

Two methods for the preparation of bioactive-PHA oligomers were developed. The first method is the transesterification of PHAs in the presence of pesticides containing a carboxyl group mediated by 4-toluenesulfonic acid monohydrate. This one-pot synthesis method is promising from a scale-up perspective because of the relatively low cost of reagents and the rather simple procedure. The second method is a two-step method; in the first step, cyclic oligomers were obtained from PHB. The second step featured the reaction of cyclic oligomers with pesticides containing the hydroxyl group in the presence of lipases. The second method, despite its greater complexity, has certain advantages. The resulting cyclic oligomers have 3 to 12 repeating units, allowing the synthesis of conjugates with more defined chain lengths. The ESI-MS^2^ analysis of the obtained products, supported by ^1^H NMR analysis, confirmed that both methods led to the formation of conjugates in which bioactive compounds are covalently bonded to PHA oligomer chains.

The one-pot transesterification method led to a small amount of (3HB-*co*-4HB) oligomers that were terminated by hydroxyl and carboxyl, as well as with unsaturated and carboxyl end groups, in addition to the desired conjugates of (3HB-*co*-4HB). However, previous studies indicated that the presence of these types of side products did not affect crops during greenhouse and field bioassays [14,15].

The developed environmentally-friendly biodegradable polymeric systems should allow for the controlled delivery of selective herbicides, thereby prolonging and improving the efficacy of those widely known herbicides. The controlled release of herbicides is accompanied by the formation of environmental-friendly non-toxic degradation products of PHA carriers. Moreover, the presence of biodegradable aliphatic polymeric carriers should show a higher resistance to weather conditions and reduced volatilities, suggesting a potential reduction of the overall environmental impact of the herbicides. Furthermore, the preliminary hydrolytic degradation tests of MCPA–oligo(3HB-*co*-4HB) conjugate gave detailed insights into the hydrolysis process, allowing identification of the degradation products formed, and confirmed the release of pesticide from the conjugates studied. This revealed the potential application of those conjugates in the field of agriculture. Field bioassays of obtained bioactive-PHA oligomers are currently underway.

The presented study links together research on industrial biotechnology and polymer science, dealing with the synthesis and structural characterisation at the molecular level of biodegradable release systems of (bio)active compounds with potential agricultural application. Our report therefore shows application of transesterification of PHA as a method for the synthesis of pesticide-oligomer conjugates from natural polyesters. Additionally, since the structures of resulting conjugates and products of their degradation have been determined at the molecular level by electrospray ionisation multistage mass spectrometry (ESI-MS^n^), the research also contributes to the polymer mass spectrometry.

Further studies on the application of these transesterification methods to other bioactive compounds containing carboxyl or hydroxyl functionalities are underway in our laboratory and will be published soon.
